# Mediating effect of self-efficacy on the relationship between social support and self-management behaviors among patients with knee osteoarthritis: a cross-sectional study

**DOI:** 10.1186/s12877-022-03331-w

**Published:** 2022-08-02

**Authors:** Yi-Yi Chen, Li-Chueh Weng, Yang-Tzu Li, Hsiu-Li Huang

**Affiliations:** 1 Department of Preventive Healthcare and Community Medicine, Taipei Medical University Hospital, Taipei, Taiwan; 2 School of Nursing, College of Medicine, Chang Gung University, Taoyuan City, Taiwan; 3 Department of Long Term Care, College of Health Technology, National Taipei University of Nursing and Health Science, Taipei, Taiwan

**Keywords:** Knee osteoarthritis, Self-efficacy, Social support, Self-management behaviors

## Abstract

**Background:**

Good self-management behaviors in patients with knee osteoarthritis can improve disease awareness, treatment effectiveness, quality of life, and reduce medical costs. However, there is a paucity of studies focusing on patients with knee osteoarthritis. Therefore, the purpose of this study was to explore the mediating effect of self-efficacy on aspects of social support and self-management behaviors in this population.

**Methods:**

This study employed a cross-sectional design and convenience sampling to survey patients with knee osteoarthritis in an outpatient department of a regional hospital in northern Taiwan from February 22, 2021, to April 15, 2021. The inclusion criteria for patients were (1) those diagnosed by a physician with knee osteoarthritis and (2) who could communicate in Chinese or Taiwanese. Participants were asked to complete a demographic questionnaire, the Arthritis Self-Efficacy Scale (ASE), the Inventory of Socially Supportive Behavior (including enacted support and perceived social support), and the Arthritis Self-Management Assessment Tool (ASMAT). In addition, the Kellgren-Lawrence Grading Scale was obtained from a chart review. Data were analyzed with descriptive statistics, t-test, one-way analysis of variance, Pearson product-moment correlation, and mediation analysis.

**Results:**

A total of 140 patients met the inclusion criteria. The mean age of participants was 70.21 ± 10.84years; most (73.6%) were female. The mean total score of the ASMAT was 64.27 ± 14.84. Scores for the ASE, enacted support, and perceived social support were significantly positively correlated with ASMAT (all *p* < .001). The standardized coefficient for total effect and direct effect of perceived social support on ASMAT was 0.899 (*p* < .001) and 0.754 (*p* < .05), respectively. After introducing the ASE into the model, the indirect effect was 0.145 (*p* < .05), which indicated that ASE had a partial mediating effect on the relationship between perceived social support and ASMAT.

**Conclusion:**

Our findings might suggest that perceived social support indirectly affected ASMAT through ASE. Therefore, interventions designed to increase self-efficacy and social support could enhance self-management behaviors for patients with knee osteoarthritis.

## Background

Knee Osteoarthritis (KOA) is one of the most common chronic diseases, resulting in poor prognosis of physical function and disability. According to the United States Centers for Disease Control and Prevention, approximately 32.5 million adults are diagnosed with degenerative arthritis each year, among which 62% are women [1]. It is estimated that, by 2040, about half of the adults over the age of 65 in the United States will have some degree of arthritis, of which the knee joint will be the most common site of degeneration [2]. The symptoms of osteoarthritis usually are exacerbated with aging, which in turn causes a heavy burden on individuals and society. In particular, patients affected by severe KOA usually report joint pain with a progressive loss of function and subsequent disability, which results in a reduction of health-related quality of life and increased depression [3, 4].

According to 2019 statistics published by Taiwan’s National Health Insurance (NHI) program on medical utilization [5], the number of KOA patients treated in outpatient/emergency clinics was about 863,000 and, on average, 1 in 10 individuals were diagnosed with KOA. The NHI program reimburses healthcare providers on a point system, rather than absolute dollars, to standardize payments over time [6]. The total cost of NHI on medical utilization was 6.6 billion points in 2019, which was 880 million points more than that in 2016 [5], suggesting that the medical cost of KOA cannot be overlooked. The British National Institute for Health and Care Excellence (NIHCE) guideline for treating degenerative arthritis emphasizes weight loss, muscle endurance training, and the provision of disease care information. Other safe and effective adjuvant therapies that can improve the effect of rehabilitation have been recommended [7]. It also encourages proactive self-management intervention to address the impact of degenerative arthritis on individuals and reduce social costs [8].

Although there was evidence that perceived self-efficacy played a role in outcomes for patients enrolled in a self-management course for chronic diseases, it was not until Lorig et al. developed a scale for measuring perceived self-efficacy for individuals with arthritis that the importance of self-efficacy for persons with osteoarthritis was confirmed [9]. The self-report scale allowed for a quantitative measure of the effects of an arthritis self-management program for persons with arthritis and rheumatism. To date, the concept of self-management is widely used in caring for patients with chronic diseases, including arthritis [10].

Self-management behaviors refer to actions taken by individuals to implement medical, behavioral, psychological and emotional participation in self-care. These actions include problem solving, decision making, resource utilization, and forming partnerships with healthcare professionals to achieve a stable, comfortable, and healthy lifestyle [11]. Previous studies have recognized that good self-management behaviors could effectively improve communication between healthcare professionals and patients, ameliorate symptoms, reduce emotional stress, increase knowledge about the disease, lower medical expenses, and improve a patient’s quality of life [10, 12].

Bandura believed that behaviors are influenced by the interaction between personal factors and the environment, in which self-efficacy is regarded as the most important factor in personal cognition [13]. Self-efficacy is defined as an individual’s level of confidence toward fulfilling a particular behavioral goal, which determines whether the behavior can be carried out, how much effort is required in the process, and how long it will persist in the event of setbacks. Previous studies have suggested that patients with higher self-efficacy may put more effort into performing self-management behaviors [14, 15]. However, human behavior is not only driven by an individual’s intrinsic self-efficacy but is also influenced by the external environment.

Social support is an external factor that is crucial to behavior maintenance and change [13]. Barrera divided social support into three categories according to different dimensions: social embeddedness, enacted support, and perceived social support [16]. Social embeddedness refers to the size, density, and degree of connection of an individual’s social network. Enacted support refers to the specific actions provided by supporters, which are also regarded as the actual acceptance of social support by individuals in need of support [16]. Perceived social support is an individual’s subjective perception of satisfaction with social support. However, enacted support does not necessarily meet an individual’s social support needs and an individual’s subjective evaluation of perceived social support is more likely to affect an individual’s health outcomes. In addition, perceived social support and enacted support are only weakly correlated; thus the two should be regarded as different dimensions of social support [17].

Geng et al. [18] found that self-efficacy and social support have a significant direct impact on cancer patients’ disease self-management, and social support can also have an indirect impact on self-management via self-efficacy and coping styles. Chan et al. [14] reported social support improved self-efficacy in patients with diabetes, thereby improving self-care behaviors. Oh and Ell [19] evaluated the association between changes in social support and self-management in patients with diabetes, which showed changes in social support were only related to self-efficacy and did not directly affect self-management. Therefore, social support might only function as a buffer in eliminating social and disease stressors.

Self-efficacy is affected by the variability of internal and external environmental resources. Most studies have suggested that self-efficacy is the essential factor that affects self-management behaviors [20]. Some studies have focused on the effect of social support and self-efficacy [14, 21] and found that social support helps patients face life challenges caused by diseases with greater confidence, thereby enhancing healthy behaviors.

Few studies have investigated the relationship between self-efficacy, social support, and self-management in patients with KOA, which are limited to exploring the effect of self-efficacy on self-management of pain control and physical activity [22–24], as well as peer support for self-management behaviors [25]. Therefore, underlying mechanisms for associations between social support, self-efficacy and overall self-management behaviors remains unclear. In addition, there is little research on how different dimensions of social support affect self-efficacy and self-management. Taiwan’s comprehensive NHI system makes it easy to quickly seek medical treatment and receive medical services when patients feel unwell or have health concerns. Due to the differences in medical care-seeking behaviors and medical resources, the self-management behaviors of Taiwanese patients may differ compared with other countries. Understanding the factors associated with self-management behaviors from the perspective of patients with KOA in Taiwan will help fill the existing knowledge gap.

Therefore, this study categorized social support dimensions into enacted support and perceived social support to explore the association between self-efficacy, social support, and self-management behavior, as well as the mediating role of self-efficacy. It is hoped that the findings of this study can provide a reference for designing appropriate care plans and measures in the future to improve the self-management behaviors and quality of life of patients with KOA.

## Methods

### Study design and setting

This was a cross-sectional study reported according to the Strengthening the Reporting of Observational Studies in Epidemiology (STROBE) recommendations [26]. The study was performed in an outpatient department of a regional hospital in northern Taiwan from February 22, 2021, to April 15, 2021.

### Participants and procedure

Patients with KOA were recruited by convenience sampling from an outpatient department of a regional hospital in northern Taiwan. The inclusion criteria for patients were (1) a diagnoses by a physician as having KOA and (2) could communicate in Chinese or Taiwanese. Patients were excluded for any of the following: (1) the presence of non-degenerative arthritis in the knees; (2) diagnosed with dementia or communication disorders; and (3) those who underwent any inpatient surgery within the past six months.

The sample size was estimated using G-power version 3.1, assuming a power of 0.80, and the α level set at 0.05. The effect size was set at a minimum value of 0.19 in reference to the correlation coefficient of 0.19 between social support and self-management behaviors in patients with rheumatoid arthritis [27]. The research framework of this study included nine independent variables and an estimated sample size of 92.

 The study objective, research process, and interviewees’ rights were explained to potential participants in detail after initiating contact. After providing informed consent, participants were asked to fill out questionnaires; if they were unable to complete the questionnaires due to illiteracy, blurred vision, or other factors, the investigator read the questions one by one to the participant and then recorded the participant’s choice for the answer. The questionnaires were coded to maintain anonymity and protect patient privacy.

### Outcome variables

The primary outcome was self-management behaviors. We used the Arthritis Self-Management Assessment Tool (ASMAT), developed by Oh et al. [11], to evaluate the self-management behaviors of patients with KOA. The scale has three core components about behaviors related to disease self-management: medical, which is comprised of 10 questions regarding adherence to treatment; behavior, which is comprised of 13 questions regarding adoption of general and healthy behaviors; and psycho-emotional, which is comprised of 9 questions about managing arthritis-induced depression, emotional problems and stress. Participants answer each question on a 4-point Likert scale from “never” = 0 points to “always” = 3 points. Total scores range from 0 to 96 points; higher scores indicate more self-management behaviors. The Cronbach’s α of the original scale was 0.88. The Chinese version of the scale was independently translated by two bilingual researchers from different fields and subsequently back translated and revised. The content validity of the revised scale was tested by clinical healthcare experts, and the overall CVI of the questionnaire was 0.97. The Cronbach’s alpha was 0.91 for the overall self-management scale and 0.86, 0.73, and 0.89 for various scales of medical management, behavior management, and psycho-emotional management, respectively.

The secondary outcomes were the Arthritis Self-Efficacy (ASE) and the socially supportive behaviors. The Arthritis Self-Efficacy (ASE) scale, developed by Lorig et al. [9], was used to assess participant’s level of confidence toward practicing specific behavioral goals. The 20-item scale has three components: self-efficacy for pain (5 items), self-efficacy for physical function (9 items), and self-efficacy for other symptoms (6 items). Each item is phrased as a statement and a visual analogue scale is used for the participant’s response, which ranges 0 points for “very uncertain” to 10 points for “very certain”; the total score is 200 points, with higher scores indicating greater self-efficacy. This study used the ASE translated into Chinese by Chen [28] and validated with 129 patients with rheumatoid arthritis in Taiwan. The Cronbach’s alpha for the total scale was determined to be .93, and the Content Validity Index (CVI) was .95 [28]. The Cronbach’s alpha for this study was .94.

Socially supportive behaviors were assessed with the 70-item Inventory of Socially Supportive Behavior (ISSB) instrument, developed by Barrera [29] and translated and edited for use in Taiwan by Yeh [30]. The ISSB is a self-report instrument developed to evaluate an individual’s satisfaction with actual enacted support (60 items) and perceived social support (10 items) provided by six social support networks: spouses, children, relatives, friends, hospital friends, and healthcare professionals. The subscale of enacted support is comprised of 10 questions each for the six support networks about the amount and frequency of support provided. Each question is scored on a 3-point Likert scale: 1 point = never; 2 points = occasionally; and 3 points = often. If any social support network is missing, the item is not scored. Scores range from 0 points, indicating no social support to 180 points indicating high social support from all six networks.

### Potential confounders

Potential confounders included participants’ characteristics and Level of knee degeneration. Demographic characteristics were collected with a questionnaire, which included gender, age, educational level, presence of other chronic diseases, the age at which the disease was diagnosed, and whether the patient had received intra-articular injection therapy, surgery, physical therapy, or analgesic medications. Body mass index (BMI) was calculated by the investigator by measuring the participant’s height and weight. Level of knee degeneration was based on the Kellgren-Lawrence Grading Scale (K-L GS), which was obtained from review of the patient’s chart. The K-L GS was evaluated by an orthopedic physician using X-ray images and data extracted from medical records. The degree of knee degeneration is then categorized into four levels, from 0 (not severe) to 4 (severe). The higher the level, the more severe the degeneration [31].

### Statistical Analysis

Data processing and analysis were performed using software package SPSS for Windows version 21.0. Statistical analysis methods included descriptive statistics (mean ± SD (standard deviation) and frequency), t-test, one-way analysis of variance, and Pearson product-moment correlation. We employed the SPSS PROCESS procedure to test the mediation model with the model-4 setup. The bias-corrected bootstrapping procedure for 5,000 repeated samplings was used to determine the 0.95 confidence interval (CI). If the CI did not contain zero, the mediating effect was considered significant; if the CI contained 0, the mediating effect was not considered significant [32].

## Results

**Fig. 1:**
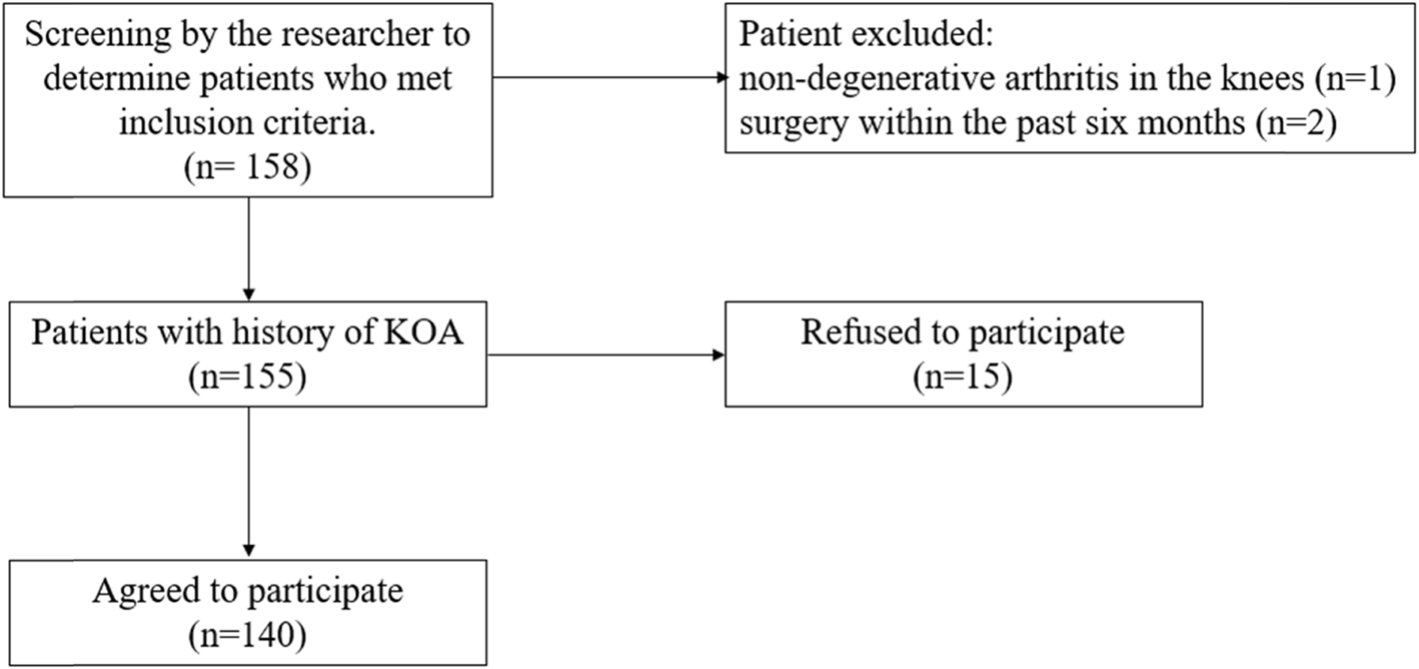
Participant recruitment

During the study period, 158 potentially eligible patients who met the inclusion criteria were seen in the outpatient clinic and 155 of those were contacted. Among them, 15 patients declined to participate in the study due to their unavailability; consequently, 140 patients who met the inclusion criteria participated in this study (Fig. 1).

Demographic and clinical characteristics of participants are shown in Table 1. The mean age of participants was 70.21 ± 10.84 years, and most were female (73.6%). The mean BMI was 25.37 ± 4.11 kg/m^2^, mean time since diagnosis was 5.94 ± 5.59 years and most had a grade of level 2 (48.6%) or level 3 (31.4%) for knee degeneration based on the K-L GS. More than 90% of participants had received an intra-articular injection, 35.7% had received knee joint surgery, 40% had received physical therapy, and 44.3% were taking analgesics.

**Table 1:** Demographic and clinical characteristics of participants (*N* = 140)

Variable	n	%	Mean	SD	Range
Age, years			70.21	10.84	29–96
Gender					
Male	37	26.4			
Female	103	73.6			
BMI, kg/m^2^			25.37	4.11	14.8–36.5
Education					
≤ Elementary school	46	32.8			
Junior/Senior High school	70	50.0			
College or above	24	17.2			
Disease duration (years)			5.94	5.54	0.1–30
Other chronic diseases					
No	40	28.6			
Yes	100	71.4			
Taking analgesics					
No	78	55.7			
Yes	62	44.3			
Physical therapy					
No	84	60			
Yes	56	40			
Intra-articular injection					
No	12	8.6			
Yes	128	91.4			
Knee joint surgery					
No	90	64.3			
Yes	50	35.7			
Disease severity (K-L GS)			2.52	0.81	1–4
Level 1	9	6.4			
Level 2	68	48.6			
Level 3	44	31.4			
Level 4	19	13.6			

*SD* standard deviation, *BMI *Body Mass Index, *K-L GS *Kellgren-Lawrence Grading Scale

Scores for the self-report instruments are shown in Table 2. The mean total score on the ASMAT was 64.27 ± 14.84 points, suggesting a moderate level of self-management. We used the metrics of % Max for component scores on the ASMAT to determine the highest and lowest overall score, which was highest for medical management (70.17%) and lowest for behavior management (64.64%). The scores on the ASE ranged from 21 to 200 points, with a mean score of 145.90 ± 39.77 points. Metrics indicated the component of functional self-efficacy was the highest score (79.7%), and pain control self-efficacy was lowest (63.14%). The mean score for perceived social support was 26.02 ± 4.58 points, indicating perceived level of social support was high (range = 12–30). The mean score for perceived enacted support was 93.92 ± 27.47 points, indicating social support from the six support networks was moderate (range = 0–180)

**Table 2:** Scores for perceived self-efficacy, social support and self-management behaviors for participants (*N* = 140)

Self-report instrument	Mean	SD	Range	% Max
Self-management score (ASMAT)	64.27	14.84	8–96	
Component scores				
Medical	21.05	5.77	6–30	70.17
Psycho-emotional	18.01	5.96	0–27	66.70
Behavior	25.21	5.67	1–39	64.64
Self-efficacy score (ASE)	145.9	39.77	21–200	
Component scores				
Physical function	71.73	18.25	10–90	79.70
Other symptoms	42.59	14.22	0–60	70.98
Pain	31.57	12.25	2–50	63.14
Social support (ISSB subscales)				
Enacted support	93.92	27.47	0- 180	
Perceived social support	26.02	4.58	12–30	

*SD *standard deviation, *% Max *(Mean÷Maximum Scale Score) X 100%, *K-L GS *Kellgren-Lawrence Grading Scale, *ASMAT *Arthritis Self-Management Assessment Tool, *ASE* arthritis self-efficacy scale, *ISSB* Inventory of Socially Supportive Behaviors

Correlation analysis between demographic characteristics and the ASMAT indicated educational level was significantly correlated (*F* = 14.28, *p* < .001). Post-hoc analysis showed correlations were lower for participants with an educational level ≤ elementary school, compared with participants who were junior/senior high school graduates, and ≥ college. Participants with other chronic diseases had a significantly lower self-management behavior score compared with those without other chronic diseases (*t* = 2.37, *p* < .01). Age (*r* = − .23, *p* < .01) and the level of arthritis (*r* = − .276, *p* = .001) were significantly negatively correlated with self-management behaviors, indicating that older age and greater severity of osteoarthritis was a barrier to self-management behaviors. Self-efficacy scores were positively correlated with self-management behaviour scores (*r* = .446, *p* < .001). In addition, both enacted support and perceived social support scores were positively correlated with total scores for self-management behaviors (*r* = .438, *p* < .001 and *r* = .310, *p* < .001, respectively), indicating the better the self-efficacy, enacted support, and perceived social support, the higher the scores for self-management behaviors (Table 3).

**Table 3:** Correlations between self-management behaviors and demographics, clinical characteristics, self-efficacy and social support for participants (*N* = 140)

		Self-management behaviors			
**Variable**	**Mean (SD)**	**F/t**	***p***	**r**	***p***
Gender		0.48	0.63		
Male	65.28 (15.10)				
Female	63.91 (14.80)				
Education		14.28	**< 0.001**		
(1) ≤ Elementary school	55.76 (15.59)				
(2) Junior/Senior high school	66.77 (12.57)				
(3) College or above	72.15 (11.89)				
**Post hoc test**		(1) < (2), (3)			
Other chronic diseases		2.37	**0.019**		
No	68.90 (15.43)				
Yes	62.42 (14.26)				
Disease duration (years)				− 0.10	0.235
Disease severity (K-L GS)				**− 0.28**	**0.001**
Other chronic diseases		2.37	**0.019**		
No	68.90 (15.43)				
Yes	62.42 (14.26)				
Taking analgesics		0.47	0.640		
No	64.80 (15.57)				
Yes	63.61 (13.96)				
Physical therapy		-1.34	0.183		
No	62.91 (15.75)				
Yes	66.32 (13.24)				
Intra-articular injection		0.15	0.882		
No	64.89 (11.76)				
Yes	64.22 (15.13)				
Knee joint surgery		− 0.38	0.702		
No	63.91 (15.92)				
Yes	64.92 (12.80)				
Age				− 0.23	**0.007**
BMI				− 0.15	0.084
Disease duration (years)				− 0.10	0.235
Disease severity (K-L GS)				**− 0.28**	**0.001**
Self-efficacy				**0.446**	**< 0.001**
Enacted support				**0.438**	**< 0.001**
Perceived social support				**0.310**	**< 0.001**

*SD* standard deviation, *BMI *Body Mass Index, *K-L GS *Kellgren-Lawrence Grading Scale

This study used the PROCESS macro version by Hayes [33] to analyse the mediating effect between self-management behaviors and perceived and enacted social support (Tables 4 and 5, respectively). Path 1 shows perceived social support (β = 0.90, *p* < .001, Table 4) and enacted support (β = 0.22, *p* < .001, Table 5) had significant explanatory power for self-management behaviors after controlling for demographic variables. Path 2 shows perceived social support had significant explanatory power for self-efficacy (β = 1.45, *p* = .046, Table 4); however, enacted support had no significant effect on self-efficacy (β = 0.12, *p* = .315, Table 5). Path 3 (Table 4) considers the explanatory power of both perceived social support and self-efficacy (β = 0.76, *p* = .003 and β = 0.10, *p* = .001, respectively) for self-management behavior as well as the explanatory power of enacted support and self-efficacy (β = 0.21, *p* < .001 and β = 0.10, *p* < .001, respectively; Table 5). In summary, all three variables had significant explanatory power for self-management behaviors.

**Table 4:** Mediation analysis of self-management behaviors and perceived social support for participants (*N* = 140)

	Self-management behaviors						Self-efficacy		
	**Model 1**			**Model 3**			**Model 2**		
**Variable**	***β***	***SE***	***p***	***β***	***SE***	***p***	***β***	***SE***	***p***
Perceived social support	0.90	0.26	< 0.001	0.76	0.25	0.003	1.45	0.72	0.046
Self-efficacy				0.10	0.03	0.001			
Gender	1.20	2.74	0.661	1.07	2.64	0.685	1.31	7.68	0.864
Age	− 0.13	0.12	0.285	− 0.08	0.12	0.489	− 0.49	0.34	0.156
K-L GS	-1.74	1.52	0.254	-1.22	1.47	0.408	-5.16	4.25	0.226
BMI	− 0.58	0.31	0.063	− 0.37	0.30	0.223	-2.05	0.86	0.019
Disease duration	− 0.05	0.22	0.832	− 0.002	0.21	0.990	− 0.43	0.60	0.476
Other diseases	0.28	2.77	0.921	0.39	2.67	0.885	-1.12	7.76	0.885
Education^a^									
Junior or Senior high school	7.89	2.69	0.004	6.31	2.63	0.018	15.93	7.53	0.036
College and above	13.11	3.72	< 0.001	10.84	3.65	0.004	22.72	10.41	0.031
		***R***^**2**^ **= 0.28**			***R***^**2**^ **= 0.34**			***R***^**2**^ **= 0.21**	
		**F = 5.63**	**< 0.001**		**F = 6.56**	**< 0.001**		**F = 3.93**	**< 0.001**
	**Effect**	***SE***	***p***	**95% CI**		**%**^**b**^			
**Total effect**	0.899	0.26	< 0.001	0.390	1.410	100			
**Direct effect**	0.754	0.25	0.003	0.255	1.254	83.87			
**Indirect effect**	0.145	0.09		0.002	0.360	16.13			

*SE *standard error, *β *Standardized coefficient, *K-L GS *Kellgren-Lawrence Grading Scale, *BMI *Body Mass Index, *CI* confidence interval

^a^ Reference: Elementary school

^b ^Percentage of effect/total effect

**Table 5:** Mediation analysis of self-management behaviors and enacted social support for participants (*N* = 140)

	Self-management behaviors						Self-efficacy		
	**Model 1**			**Model 3**			**Model 2**		
**Variable**	***β***	***SE***	***p***	***β***	***SE***	***p***	***β***	***SE***	***p***
Enacted social support	0.22	0.04	< 0.001	0.21	0.04	< 0.001	0.12	0.12	0.315
Self-efficacy				0.10	0.03	< 0.001			
Gender	− 0.66	2.59	0.799	− 0.64	2.48	0.685	− 0.19	7.80	0.981
Age	− 0.10	0.12	0.379	− 0.05	0.11	0.489	− 0.47	0.35	0.176
K-L GS	-1.76	1.42	0.218	-1.17	1.37	0.408	-5.80	4.28	0.178
BMI	− 0.63	0.29	0.031	− 0.43	0.28	0.223	-1.93	0.87	0.028
Disease duration	− 0.20	0.20	0.324	− 0.12	0.19	0.990	− 0.70	0.59	0.239
Other diseases	− 0.83	2.58	0.749	− 0.50	2.47	0.885	-3.19	7.77	0.683
Education^a^									
Junior or Senior high school	8.01	2.53	0.002	6.37	2.46	0.011	16.02	7.62	0.037
College and above	9.37	3.57	0.010	7.25	3.46	0.038	20.67	10.73	0.056
		***R***^**2**^ **= 0.36**			***R***^**2**^ **= 0.42**			***R***^**2**^ **= 0.20**	
		**F = 8.16**	**< 0.001**		**F = 9.40**	**< 0.001**		**F = 3.52**	**< 0.001**
	**Effect**	***SE***	***p***	**95% CI**		**%**^**b**^			
**Total effect**	0.217	0.040	< 0.001	0.139	0.296	100			
**Direct effect**	0.205	0.038	< 0.001	0.130	0.280	94.47			
**Indirect effect**	0.012	0.013		− 0.013	0.040	5.53			

*Abbreviations: SE *standard error, *β *Standardized coefficient, *K-L GS *Kellgren-Lawrence Grading Scale, *BMI *Body Mass Index, *CI *confidence interval

^a^ Reference: Elementary school

^b ^Percentage of effect/total effect

The standardized coefficient for the total effect and direct effect of perceived social support on self-management behavior was 0.899 (95% CI: 0.390, 1.410) and 0.754 (95% CI: 0.255, 1.254), respectively (Table 4). After introducing the mediator variable of self-efficacy into the model, the standardized coefficient of the indirect effect was 0.145 (95% CI: 0.002, 0.360), which was statistically significant, indicating that self-efficacy had a partial mediating effect, accounting for 16.13% of the total effect. As shown in Table 5, the standardized coefficient for the total effect of enacted support on self-management behavior was 0.217 (95% CI: 0.139, 0.296), and the direct effect was 0.205 (95% CI: 0.130, 0.280). After introducing the mediator variable of self-efficacy into the model, the standardized coefficient of the indirect effect was 0.012 (95% CI: − 0.013, 0.040), which was not significant, indicating that enacted support directly affected self-management behaviors with no mediating effect from self-efficacy.

## Discussion

This study aimed to explore the mediating effect of self-efficacy on aspects of social support and self-management behaviors in patients with KOA. Our findings showed enacted support, perceived social support and self-efficacy, were significantly positively correlated with self-management behaviors for individuals with KOA. Self-efficacy had a partial mediating effect on the relationship between perceived social support and self-management behaviors. We also found older age and greater severity of KOA were barriers to self-management behaviors.

 Scores on the ASMAT indicated participants in our study had a moderate level of self-management behaviors, with scores for medical management highest, and behavior management lowest. The content of the medical management component included questions about whether the participant saw a physician on a regular basis, was compliant about taking prescribed medications, and discussions of treatment plans and medical decisions with healthcare professionals, which suggests participants took responsibility for managing their personal healthcare. The component of behavior management included questions about whether the participant sought healthcare information, attended disease and healthcare seminars, and used non-prescription treatments to reduce pain, such as massage. The low scores for behavior management suggest this area of healthcare was not an important part of participants’ self-management. Due to the successful implementation of the NHI system in Taiwan, the public has easy access to a doctor, low medical expenses, and no barriers to periodic follow-ups or obtaining a prescription, which may explain why medical self-management behaviors scored high. At the same time, the NHI system may explain the low scores for behavior management. The easy access to healthcare allows individuals to seek medical treatment as their first choice when addressing health problems, thus they may not consider daily self-management behaviors to be as important.

Patient age, education level, comorbidities, and the severity of arthritis were significantly associated with self-management behaviors, which echo previous research. Older patients may have reduced muscle strength and joint mobility due to aging. As the prevalence of multiple chronic diseases increases with age, people often lower their expectations of their ability to perform physical activities, which can result in decreased self-efficacy that subsequently affects self-management behaviors [15]. Kang et al. [12] indicated that the self-management of patients with multiple chronic diseases is relatively complex, resulting in poor self-management behaviors. Patients with severe joint degeneration may be more dependent on osteoarthritis medications and thus have poorer self-management behaviors [34]. Consistent with previous research findings, this study found that patients with higher education levels were more likely to actively seek disease-related knowledge and resources and discuss their conditions and treatment plans with healthcare professionals [35].

In this study, self-efficacy was positively correlated with scores for self-management behaviors, implying that when patients had higher confidence toward performing self-management activities, they were more likely to implement self-management behaviors. Previous correlation studies on patient groups, such as those with diabetes and coronary heart disease, had similar results [21, 36]. Improving self-efficacy is an important intervention in clinical care. Healthcare workers can provide information on arthritis care and individualized guidance, encourage and assist in solving individual problems, and create successful experiences related to implementing self-management behaviors [15]. Patients can also be encouraged to join supportive groups and utilize observational learning and vicarious learning skills to discuss health problems caused by disease and coping methods to reduce anxiety or negative emotions [37]. Patients can also discuss specific methods that can successfully relieve pain and delay disability to help improve self-efficacy and enhance self-management behaviors.

Our findings are in line with previous research showing that social support is positively correlated with self-management behaviors [14, 38]. Healthcare workers can formally play a social support role by partnering with patients to provide care information about the disease, elevate their sense of support, and integrate social support into the self-management program curriculum. In addition, healthcare workers can provide patients with an appropriate environment to discuss their conditions or problems, such as follow-ups by phone or software apps to ensure that patients have a supportive environment, a good social network, and support resources. All these measures can promote patients’ physical activities and develop self-management behaviors, which also help reduce the frequency of doctor visits and encourage the efficient use of healthcare resources to reduce healthcare costs and achieve self-management goals [14, 39].

This study investigated the role of self-efficacy in the relationship between enacted support, perceived social support, and self-management behaviors. The results showed that self-efficacy partially mediated the relationship between perceived social support and self-management behaviors but had no significant mediating effect on the relationship between enacted support and self-management behaviors. Perceived social support and self-efficacy are intrinsic psychological resources that enable individuals to regulate their thought process and behavior. According to the self-efficacy theory, an individual’s self-efficacy for healthy behavior may depend in part on psycho-emotional states. A recent study found a positive motivation for achievement influenced older adults’ self-efficacy in their willingness to be reemployed [40]. By contrast, patients troubled by pessimism and emotions often lose confidence in their ability to perform self-management behaviors [37]. On the other hand, perceived social support may be influenced by self-efficacy through psychological factors, enabling patients to overcome their problems by receiving encouragement and emotional and information support. Therefore, greater perceived social support can strengthen personal self-efficacy and indirectly enhance self-management behaviors [14]. Conversely, when a patient’s perceived social support is insufficient, their confidence in self-management behavior may decrease [21]. In this study, enacted support had a direct effect on self-management behavior without the mediation of self-efficacy. Healthcare workers s are one of the important social support networks for patients, and they can put forth practical action to support and enhance patients’ self-management behaviors. By establishing partnerships with patients, they can also strengthen patients’ perceived social support. They can employ strategies that increase self-efficacy to achieve the goal of enhancing patients’ self-management behaviors and improving quality of life.

### Study Limitations

Due to time and labor considerations, all participants were recruited from one regional hospital in Taipei City. Therefore, the findings of this study may not be used to infer the self-management behaviors of patients with KOA at different medical institutions in Taiwan. The cross-sectional design of this study only revealed the self-management status of patients at a particular point in time; however, KOA is a long-term chronic disease with a high risk of disability. Thus, it is suggested that use of the International Classification of Functioning, Disability, and Health (ICF) [41] and tracking the long-term changes in self-management behaviors of individuals with KOA be considered for future research.

## Conclusion

The results of this study support the association between self-efficacy, social support, and self-management behavior and demonstrated that perceived social support indirectly affected self-management behavior through self-efficacy. In terms of clinical practice, healthcare workers should first evaluate patients’ self-management behaviors and focus on social support and self-efficacy intervention strategies to provide the necessary social support and resources in any insufficient areas. Physicians should help increase patients’ perceived social support and self-efficacy and promote the implementation of self-management behaviors. Enacted support directly affected self-management behaviors without the mediating effect of self-efficacy. Healthcare workers are one of the essential social support networks for patients. They can provide active support to enhance patients’ self-management behaviors. By establishing partnerships with patients, healthcare workers can strengthen patients’ perceived social support and implement strategies designed to improve self-efficacy to achieve the goals of enhancing patients’ self-management behaviors and improving their quality of life.

## Data Availability

The datasets used and/or analysed during the current study are available from the corresponding author on reasonable request.
